# Supplementary material for: Identification and Characterization of Novel SPHINX/BMMF-like DNA Sequences Isolated from Non-Bovine Foods
Source: Genes (Basel). 2023 Jun 21;14(7):1307. doi: 10.3390/genes14071307 (PMC10378824; doi:10.3390/genes14071307)
Supplement: Supplementary file 1 [file genes-14-01307-s001.zip › FiguresS1-3.pdf]

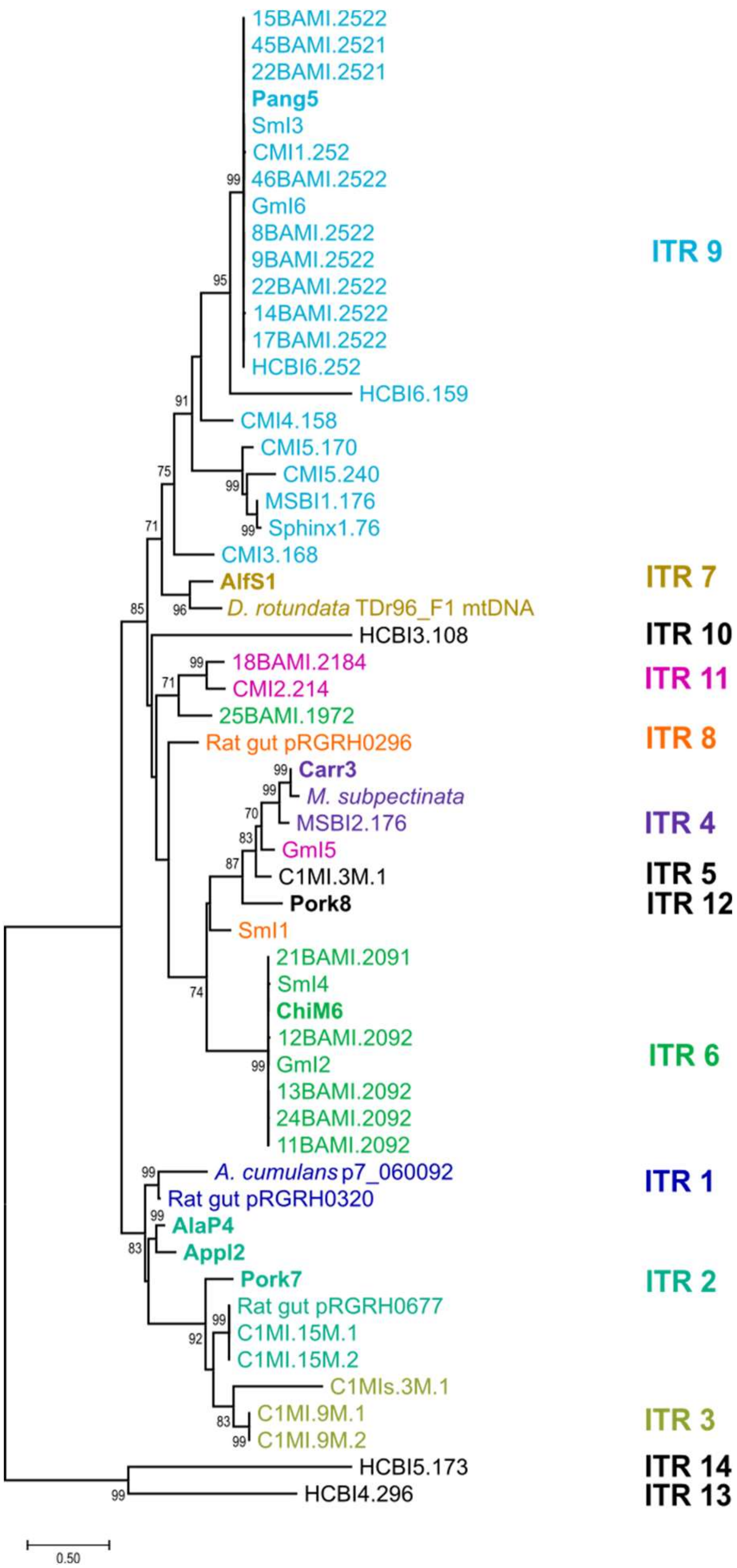

Figure S1:

Phylogenetic tree of full-length SPHINX/BMMF group 1 sequences. The evolutionary history was inferred by using the Maximum Likelihood method and Hasegawa-Kishino-Yano model [20]. From 250 bootstrap replications the tree with the highest log likelihood (-46133.86) is shown. Bootstrap values above 70% are shown next to the branches. A discrete Gamma distribution was used to model evolutionary rate differences among sites (5 categories (+*G*, parameter = 0.7580)). The tree is drawn to scale, with branch lengths measured in the number of substitutions per site. This analysis involved 56 nucleotide sequences. There were a total of 3277 positions in the final dataset. Names are colored according to the associated ITR motif (see Figure 2b).

a)

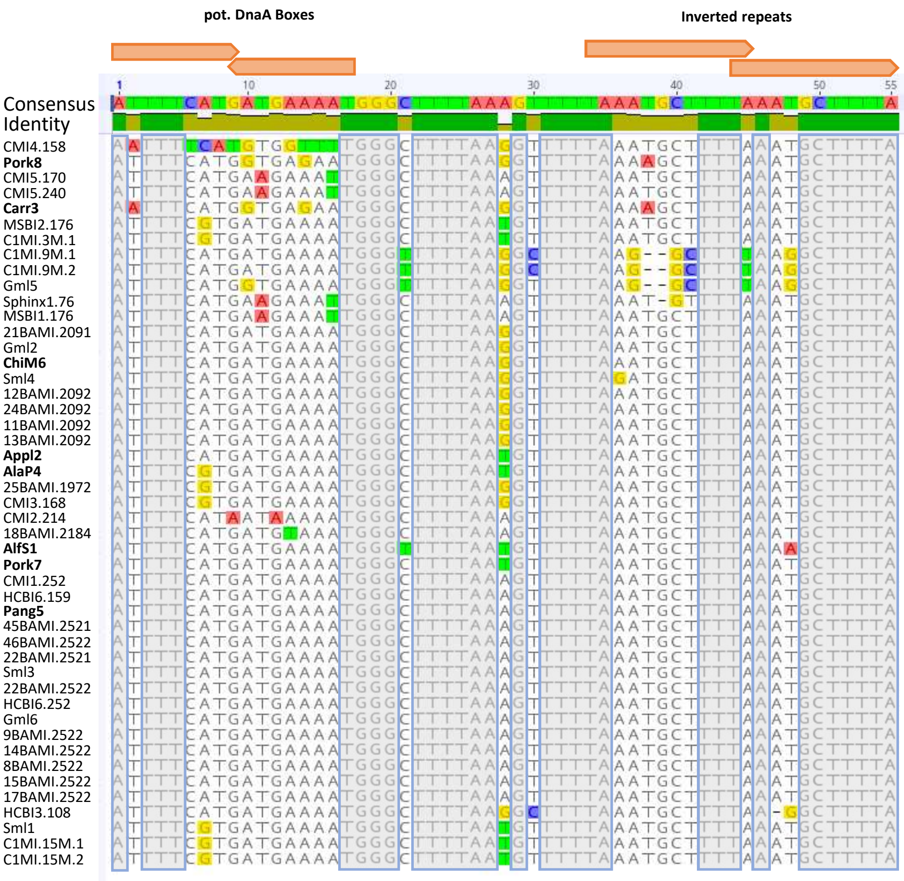

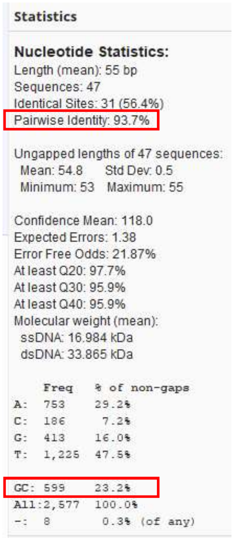

b)

Yersinia pestis PD1THRMRP	TTATCCAAA
Pseudomonas syringae X58896	ATATCCACA
Pseudomonas alcaligenes Y10829	TTTTCCACA
Uncultured Bacteria AJ851089-1	TTATCCACT
Uncultured Bacteria AJ851089-2	TTATCCACG
Pseudomonas sp. AJ421512-1	TTACCCAAT
Pseudomonas sp. AJ421512-2	TTATTCCCG
Seratia sp. AF468972	TTATCCACA
Buchnera aphidicola LR217697	TTATGCACC
Buchnera aphidicola DQ114495	TTATCCACA
Buchnera aphidicola LR025087	TTATCCACC
Pseudomonas aeruginosa AY257539-1	TTATCCCCA
Pseudomonas aeruginosa Y257539-2	CTATCCACA
APseudomonas aeruginosa Y257539-4	TTATCCACG
Buchnera aphidicola LR217694	TTATGCACA
Buchnera aphidicola LR217702-12	TTATGCATA
Buchnera aphidicola LR217702-13	CTATCCATA
Putative DnaA Box in Sphinx/BMMF group 1	ATTTTCATG

Deduced consensus Sequence

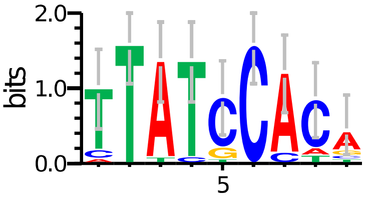

Figure S2:

a) ClustalW alignment of 47 SPHINX/BMMF group 1 sequences, differences are colored, frames show identical regions, potential DnaA-Boxes (described in b) and inverted repeats are indicated by orange arrows, pairwise identity and GC-content are framed in red.

b) ClustalW alignment of 17 sequences of DnaA-Boxes extracted from NCBI Nucleotide database. Deduced consensus sequence illustrated using WebLogo tool.

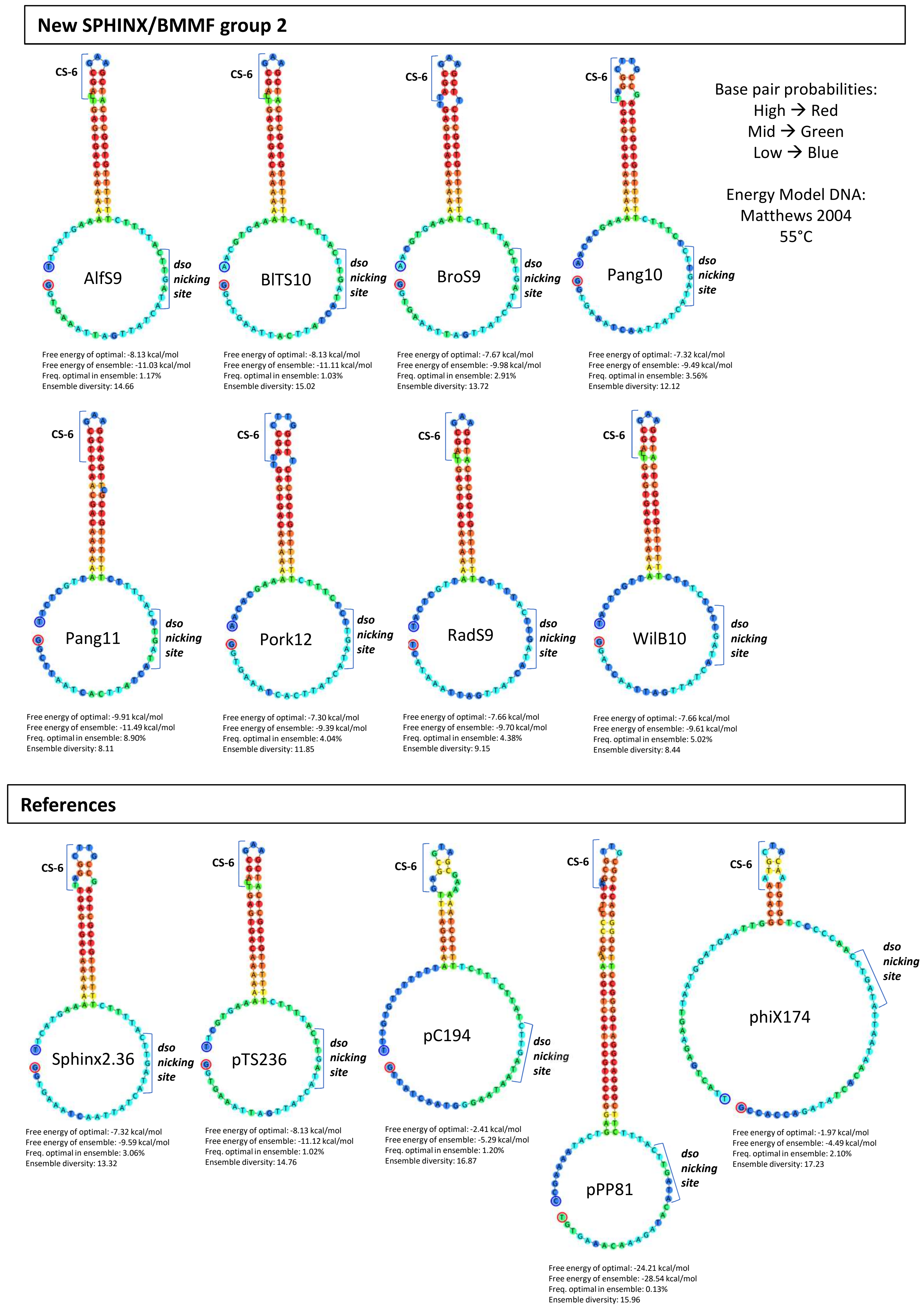

Figure S3: DNA Fold calculation, *dso* nicking site and *sso* CS-6 site
