# Supplementary material for: Identification and Characterization of Novel SPHINX/BMMF-like DNA Sequences Isolated from Non-Bovine Foods
Source: Genes (Basel). 2023 Jun 21;14(7):1307. doi: 10.3390/genes14071307 (PMC10378824; doi:10.3390/genes14071307)
Supplement: Supplementary file 1 [file genes-14-01307-s001.zip › TableS3.docx]

**Table S3**: Orffinder and Blastp analysis of SPHINX/BMMF group 1 and 2 sequences

| **Blastp analysis, nr_protein database** | | |
| --- | --- | --- |
| Name | Blastp result | Identity |
| **S/B group 1** | | |
| AlfS1 ORF 1 | replication initiation protein RepM [Acinetobacter junii] WP_129264380 | 99,69% (324aa/324aa target) |
| Carr3 ORF 1 | replication initiation protein RepM [Campylobacter helveticus] WP_139022403 | 97,49% (319aa/319 target) |
| AlaP4 ORF 1 | replication initiation protein [Salmonella enterica subsp. enterica serovar Agona] EBL5619743 | 95,62% (324aa/325aa target) |
| Pang5 ORF 1 | replication protein [BMMF1 DNA sequence] QWQ66138 | 100% (326aa/326 target) |
| Pang5 ORF 2 | MULTISPECIES: hypothetical protein [Acinetobacter] WP_213073224 | 100% (219aa/219 target) |
| ChiM6 ORF 1 | replication protein [BMMF1 DNA sequence] QWQ66128 | 99,96% (327aa/327 target) |
| ChiM6 ORF 2 | hypothetical protein [BMMF1 DNA sequence] UEJ84713 | 99,96% (106aa/106 target) |
| Pork7 ORF 1 | MULTISPECIES: replication initiation protein RepM [Acinetobacter] WP_168379861 | 100% (322aa/319 target) |
| Pork7 ORF 2 | hypothetical protein, partial [Acinetobacter gerneri] WP_042073725 | 58,44% (100aa/77 target) |
| Pork8 ORF 1 | unnamed protein product [Brugia timori] VDO15958 | 94,59% (297aa/318 target) |
| Appl2 ORF 1 | replication initiation protein [Salmonella enterica subsp. enterica serovar Agona] EBL5619743 | 99,69% (328aa/325 target) |
| **S/B group 2** | | |
| AlfS9 ORF 1_a | protein rep, partial [Acinetobacter baumannii] WP_188199462 | 96,85% (129aa/196 target) |
| AlfS9 ORF 1_b | replication protein, partial [BMMF2 DNA sequence] VEV85753 | 99,03% (319aa/435 target) |
| AlfS9 ORF 2 | hypothetical protein [Acinetobacter ursingii] WP_151788947 | 100% (107aa/107 target) |
| AlfS9 ORF 3 | uncharacterized protein, partial [BMMF2 DNA sequence] VEV85747 | 87,50% (112 aa/112 target) |
| RadS9 ORF 1 | replication protein, partial [BMMF2 DNA sequence] VEV85753 | 100% (435aa/435 target) |
| RadS9 ORF 2 | hypothetical protein [Janibacter anophelis] WP_205353127 | 98,23% (113aa/113 target) |
| RadS9 ORF 3 | uncharacterized protein, partial [BMMF2 DNA sequence] VEV85474 | 100% (96aa/96 target) |
| BroS9 ORF 1_a | protein rep, partial [Acinetobacter baumannii] MBE2397913 | 99,59% (255aa/293 target) |
| BroS9 ORF 1_b | protein rep, partial [Acinetobacter baumannii] MBE2785545 | 90,86% (211aa/249 target) |
| BroS9 ORF 2 | hypothetical protein ILR45_17855 [Acinetobacter baumannii] MBE2397911 | 100% (126aa/126 target) |
| BroS9 ORF 3 | MULTISPECIES: hypothetical protein [Gammaproteobacteria] WP_071851674 | 100% (96aa/96 target) |
| BltS10 ORF 1 | protein rep [Acinetobacter towneri] WP_253105181 | 98,06% (421aa/431 target) |
| BltS10 ORF 2 | uncharacterized protein, partial [BMMF2 DNA sequence] VEV85602 | 96,53% (144aa/144 target) |
| BltS10 ORF 3 | uncharacterized protein, partial [BMMF2 DNA sequence] VEV85557 | 100% (97aa/99 target) |
| Pang11 ORF 1 | protein rep [Acinetobacter puyangensis] WP_097080553 | 77,63% (438aa/439 target) |
| Pang11 ORF 2 | uncharacterized protein, partial [BMMF2 DNA sequence] VEV85570 | 97,32% (120aa/119 target) |
| Pang11 ORF 3 | hypothetical protein [Acinetobacter towneri] WP_253105183 | 94,79% (96aa/96 target) |
| Pang10 ORF 1 | protein rep [Acinetobacter indicus] WP_104499427 | 96,17% (340aa/345 target) |
| Pang10 ORF 2 | hypothetical protein [Neisseria meningitidis] WP_127245529 | 74,62% (130aa/130 taregt) |
| Pang10 ORF 3 | MULTISPECIES: hypothetical protein [unclassified Acinetobacter] WP_179991767 | 94,79% (96aa/96 target) |
| WilB10 ORF 1 | protein rep [Acinetobacter sp. SH20PTE14] WP_233945594 | 99,41% (338aa/340 target) |
| WilB10 ORF 2 | uncharacterized protein, partial [BMMF2 DNA sequence] VEV85570 | 100% (111aa/119 target) |
| WilB10 ORF 3 | uncharacterized protein, partial [BMMF2 DNA sequence] VEV85474 | 100% (96aa/96 target) |
| Pork12 ORF 1 | protein rep [Acinetobacter variabilis] WP_200231015 | 100% (340aa/340 target) |
| Pork12 ORF 2 | hypothetical protein [Acinetobacter sp. YH12073] WP_180113482 | 95,65% (145aa/145 target) |
| Pork12 ORF 3 | MULTISPECIES: hypothetical protein [unclassified Acinetobacter] WP_179991767 | 97,92% (96aa/98 target) |
| **HHPred analysis, Pfam-A_v35 database** | | |
| Name | Result, Pfam Homolog | Probability |
| **S/B group 1** | | |
| AlfS1 ORF 1 | PF01051.24; Rep_3; Initiator Replication protein | 99,98% (324aa/215aa target) |
| Carr3 ORF 1 | PF01051.24; Rep_3; Initiator Replication protein | 100 (319aa/215 target) |
| AlaP4 ORF 1 | PF01051.24; Rep_3; Initiator Replication protein | 99,97% (328aa/215 target) |
| Pang5 ORF 1 | PF01051.24; Rep_3; Initiator Replication protein | 99,98 (326aa/215 target) |
| Pang5 ORF 2 | PF01878.21; EVE; EVE domain  PF19807.2; DUF6290; Family of unknown function (DUF6290) | 98,41% (219aa/147 target)  97,5% (219aa/71 target) |
| ChiM6 ORF 1 | PF01051.24; Rep_3; Initiator Replication protein | 99,98% (327aa/215 target) |
| ChiM6 ORF 2 | PF11196.11; DUF2834; Protein of unknown function DUF2834 | 97,86% (106aa/97 target) |
| Pork7 ORF 1 | PF01051.24; Rep_3; Initiator Replication protein | 99,98% (327aa/215 target) |
| Pork7 ORF 2 | PF16050.8; CDC73_N; Paf1 complex subunit CDC73 N-terminal | 41.85% (100aa/302 target) |
| Pork8 ORF 1 | PF01051.24; Rep_3; Initiator Replication protein | 100% (297aa/215 target) |
| Appl2 ORF 1 | PF01051.24; Rep_3; Initiator Replication protein | 99,97% (328aa/215 target) |
| **S/B group 2** | | |
| AlfS9 ORF 1_a | PF01446.20; Rep_1; Replication protein | 96,4% (129aa/250 target) |
| AlfS9 ORF 1_b | PF01446.20; Rep_1; Replication protein | 99,92% (319aa/250 target) |
| AlfS9 ORF 2 | PF10723.12; RepB-RCR_reg; Replication regulatory protein RepB | 97,69% (107aa/81 target) |
| AlfS9 ORF 3 | PF17426.5; Putative_G5P; Putative Gamma DNA binding protein G5P | 98,32% (112 aa/108) |
| RadS9 ORF 1 | PF01446.20; Rep_1; Replication protein | 99,93% (435aa/250 target) |
| RadS9 ORF 2 | PF10723.12; RepB-RCR_reg; Replication regulatory protein RepB | 97,3% (113aa/81 target) |
| RadS9 ORF 3 | PF17426.5; Putative_G5P; Putative Gamma DNA binding protein G5P | 98,45% (96aa/108 target) |
| BroS9 ORF 1_a | PF01446.20; Rep_1; Replication protein | 99,8% (255aa/250 target) |
| BroS9 ORF 1_b | PF01446.20; Rep_1; Replication protein | 98,85% (211aa/250 target) |
| BroS9 ORF 2 | PF12441.11; CopG_antitoxin; CopG antitoxin of type II toxin-antitoxin system  PF10723.12; RepB-RCR_reg; Replication regulatory protein RepB | 97,96% (126aa/79 target)  97,53 (126ss/81 target) |
| BroS9 ORF 3 | PF17426.5; Putative_G5P; Putative Gamma DNA binding protein G5P | 98,43% (96aa/108 target) |
| BltS10 ORF 1 | PF01446.20; Rep_1; Replication protein | 99,94% (421aa/250 target) |
| BltS10 ORF 2 | PF03693.17; ParD_antitoxin; Bacterial antitoxin of ParD toxin-antitoxin type II system and RHH  PF10723.12; RepB-RCR_reg; Replication regulatory protein RepB | 98,42% 144aa/80 target)  97,74 (144aa/81 target) |
| BltS10 ORF 3 | PF17426.5; Putative_G5P; Putative Gamma DNA binding protein G5P | 98,94% (97aa/108 target) |
| Pang11 ORF 1 | PF01446.20; Rep_1; Replication protein | 99,94% (438aa/250 target) |
| Pang11 ORF 2 | PF10723.12; RepB-RCR_reg; Replication regulatory protein RepB | 97,58% (120aa/81 target) |
| Pang11 ORF 3 | PF17426.5; Putative_G5P; Putative Gamma DNA binding protein G5P | 98,41% (96aa/108 target) |
| Pang10 ORF 1 | PF01446.20; Rep_1; Replication protein | 99,94% (340aa/250 target) |
| Pang10 ORF 2 | PF07878.14; RHH_5; CopG-like RHH_1 or ribbon-helix-helix domain, RHH_5  PF10723.12; RepB-RCR_reg; Replication regulatory protein RepB | 97,55% (130aa/43 target)  96,92% (130aa/81 target) |
| Pang10 ORF 3 | PF17426.5; Putative_G5P; Putative Gamma DNA binding protein G5P | 98,47% (96aa/108 target) |
| WilB10 ORF 1 | PF01446.20; Rep_1; Replication protein | 99,94% (338aa/250 target) |
| WilB10 ORF 2 | PF10723.12; RepB-RCR_reg; Replication regulatory protein RepB | 97,9% (111aa/81 target) |
| WilB10 ORF 3 | PF17426.5; Putative_G5P; Putative Gamma DNA binding protein G5P | 98,45% (96aa/108 target) |
| Pork12 ORF 1 | PF01446.20; Rep_1; Replication protein | 99,94% (340aa/250 target) |
| Pork12 ORF 2 | PF12441.11; CopG_antitoxin; CopG antitoxin of type II toxin-antitoxin system  PF10723.12; RepB-RCR_reg; Replication regulatory protein RepB | 97,77% (145aa/79 target)  97,56% (145aa/81 target) |
| Pork12 ORF 3 | PF17426.5; Putative_G5P; Putative Gamma DNA binding protein G5P | 98,43% (96aa/108 target) |
